# Heterozygote Advantage of the rs3794624 Polymorphism in *CYBA* for Resistance to Tuberculosis in Two Chinese Populations

**DOI:** 10.1038/srep38213

**Published:** 2016-11-30

**Authors:** Qianqian Liu, Shouquan Wu, Miao Xue, Andrew J. Sandford, Jingcan Wu, Yu Wang, Guo Chen, Chuanmin Tao, Yin Tang, Yulin Feng, Jun Luo, Jian-Qing He

**Affiliations:** 1 Department of Respiratory and Critical Care Medicine, West China Hospital, Sichuan University, Chengdu, Sichuan, China; 2 Department of Respiratory Diseases, Chengdu Municipal First People’s Hospital, Chengdu, Sichuan, China; 3 Department of Laboratory Medicine, West China Hospital, Sichuan University, Chengdu, Sichuan, China; 4 Centre for Heart Lung Innovation, University of British Columbia and St. Paul’s Hospital, Vancouver, BC, Canada; 5 Division of Geriatrics, Sichuan Provincial People’s Hospital, Chengdu, Sichuan, China; 6 State Key Laboratory of Oral Disease, West China School & Hospital of Stomotology, Sichuan University, Chengdu, Sichuan, China; 7 Division of Infectious Diseases, People’s Hospital of Aba Tibetan Autonomous Prefecture, Maer, Sichuan, China

## Abstract

Phagocyte Nicotinamide Adenine Dinucleotide Phosphate (NADPH) oxidase complex is a key enzyme that catalyzes the production of reactive oxygen species, which mediate oxygen-dependent killing of microorganisms, such as *Mycobacterium tuberculosis*. P22phox, encoded by *CYBA*, is the key regulatory subunit of NADPH oxidase. Our study aimed to investigate the association of *CYBA* polymorphisms with susceptibility to tuberculosis. Three SNPs (rs9932581, rs3794624 and rs4673) were genotyped in the discovery cohort composed of Chinese Han individuals. We found that the A allele of rs3794624 was a significant protective factor against tuberculosis (GA vs. GG: OR = 0.74, 95% CI 0.57–0.96; GA vs. GG+AA: OR = 0.73, 95% CI 0.56–0.95), which was then replicated in the Chinese Tibetan population (GA vs. GG: OR = 0.68, 95% CI 0.51–0.92; AA+GA vs. GG: OR = 0.70, 95% CI 0.52–0.93; GA vs. GG+AA: OR = 0.68, 95% CI 0.51–0.92). Meta-analysis including both cohorts identified overdominance as the best genetic model and provided robust evidence for the protective effect of the rs3794624 GA genotype against tuberculosis without any evidence of heterogeneity (GA vs. GG+AA: OR = 0.71, 95% CI 0.58–0.86). Our study found an association between the GA genotype of rs3794624 in *CYBA* with decreased tuberculosis susceptibility in two Chinese populations. Further analyses are needed to reveal the potential function of this SNP.

Tuberculosis (TB), caused by *Mycobacterium tuberculosis* (MTB), claims 1.5 million lives annually and remains the most important public health problem in developing countries, especially China^1^. According to epidemiological data, only a small fraction (5–15%) of the population infected with MTB will develop clinically active disease during their lifetime^2^. Susceptibility to TB is likely due to variation in MTB virulence, host genetic factors as well as environmental determinants^3^,^4^,^5^. As acquired immunodeficiency syndrome and other immune-compromising conditions significantly increase the risk of developing TB, it is clear that protective immune responses function efficiently to combat the invading MTB in the majority of individuals^6^. In recent years, a large number of genetic association studies have been conducted that have mainly focused on host immunity against MTB, and the results of these studies have enhanced our understanding of the pathogenesis of TB^7^.

The innate immune system serves as the first line of defense against MTB infection that recognizes and phagocytizes invading pathogen, rapidly killing the engulfed microbe, limits its growth and proliferation, induces apoptosis, and promotes the secretion of chemokines and pro-inflammatory cytokines^6^. It is worth noting that reactive oxygen species (ROS) produced by the phagocyte Nicotinamide Adenine Dinucleotide Phosphate (NADPH) oxidase complex during the respiratory burst is suggested to play a critical role throughout the entire innate immune defenses, contributing to the clearance of MTB^8^. Dysfunction of the phagocyte NADPH oxidase complex leads to a primary immunodeficiency, chronic granulomatous disease, which exhibits an especially high risk of clinical TB and BCG complications^9^,^10^,^11^,^12^,^13^. Thus, there is a strong rationale for the investigation of phagocyte NADPH oxidase complex function with respect to TB susceptibility.

Phagocyte NADPH oxidase complex is comprised of six subunits, including two transmembranous subunits (gp91phox and p22phox), three cytosolic subunits (p67phox, p47phox, and p40phox), and a GTP-binding protein (Rac1 or 2)^14^. Of these subunits, p22phox (encoded by *CYBA*) is the key regulatory subunit that acts as the final transporter in the electron-transfer chain from NADPH to molecular oxygen^15^. However, to our knowledge no studies have evaluated the association of polymorphisms in *CYBA* with TB susceptibility, although polymorphisms in this gene have been reported to exert an influence on several ROS-associated diseases, such as hypertension, coronary heart disease and chronic obstructive pulmonary disease^16^,^17^,^18^. Therefore, the aim of our study was to determine the role of polymorphisms in *CYBA* in susceptibility to TB.

## Materials and Methods

### Study population

In the discovery cohort, a total of 1244 unrelated ethnic Han Chinese (636 TB patients and 608 healthy controls) were recruited between July 2012 and August 2014 from the West China Hospital of Sichuan University. An independent replication cohort was composed of Chinese Tibetans (613 TB patients and 603 healthy controls) consecutively recruited between February 2013 and August 2015 from the People’s Hospital of the Aba Tibetan Autonomous Prefecture. The study protocol was approved by the ethics committee of West China Hospital and People’s Hospital of Aba Tibetan Autonomous Prefecture. Methods were carried out in accordance with the approved guidelines. Written informed consent was obtained from each subject. Demographic characteristics of all participants were collected from a detailed questionnaire.

The diagnosis of TB was based on the following criteria: culture positive for MTB and/or smear positive for MTB and/or histopathological findings of TB disease and/or clinical and radiographic presentation consistent with TB, with positive response to anti-TB therapy. The healthy control groups were selected from individuals who presented to the outpatient department of the West China Hospital or the People’s Hospital of the Aba Tibetan Autonomous Prefecture for annual physical examination, without active TB, without history of TB and matched with cases by gender and age.

Individuals with the following conditions were excluded from the study: human immunodeficiency virus infection, autoimmune disease, cancer, primary immunodeficiency, treatment with immunosuppressive drugs, diabetes mellitus.

### SNP selection

SNPs in the region between 3,000 base pairs upstream and 2000 base pairs downstream of *CYBA* were screened based on literature review^16^,^19^,^20^ and *in silico* functional prediction from the FuncPred (http://snpinfo.niehs.nih.gov/snpinfo/snpfunc.htm) and Regulome DB (http://regulome.stanford.edu/) databases. Only those SNPs with significant disease associations and potential effects on function were included in our study. As a result, three SNPs (rs9932581 T > C, rs3794624 G > A and rs4673 T > C) were selected. rs9932581 is located in the 5’ promoter region of *CYBA*, at position −930 from the start codon, and was reported to be associated with hypertension and coronary artery disease^16^,^21^. The rs9932581 polymorphism was demonstrated by mutagenesis experiments to result in altered promoter activity. rs3794624, located in intron 1, was found to be a strong contributing factor to the Ankle-Brachial Index (p = 6.3×10^−5^) and have moderate effects on postmenopausal breast cancer risk^19^,^22^. And both above-mentioned SNPs were predicted to be potential transcription factor binding sites by the FuncPred and Regulome DB databases (rs9932581 T > C scored 4; rs3794624 G > A scored 2a). rs4673 was reported to be associated with essential arterial hypertension, coronary artery disease, and type 2 diabetes mellitus and so on^20^,^23^,^24^. It is a non-synonymous SNP located in exon 4, that leads to a histidine/tyrosine substitution and was shown to have an influence on both basal and NADH-stimulated superoxide production^20^. The FuncPred database also reported that the substitution was possibly damaging by Polyphen analysis.

### Genotyping

We collected venous blood samples (4–5 ml) in EDTA tubes (BD Vacutainers, Franklin Lakes, NJ, USA) from each subject. Genomic DNA was isolated using the AxyPrep DNA Blood kit (Axygen Scientific Inc, Union City, CA, USA), according to the manufacturer’s instructions. Genotyping of the discovery cohort was carried out by Sequenom’s iPLEX SNP genotyping protocol using matrix-assisted laser desorption/ionization time of flight mass spectrometry on the MassArray Analyzer 4 system (Sequenom Inc., San Diego, CA, USA). SNP genotyping of the replication cohort was performed using a SNPscan Kit (Cat#:G0104, Genesky Biotechnologies Inc., Shanghai, China) as described previously^25^. The SNPscan genotyping technology is based on double ligation and multiplex fluorescence polymerase chain reactions. As a quality control measure, we genotyped 5% of the samples in duplicate to check for concordance using the same method.

### Statistical analyses

Comparisons of the demographic characteristics between cases and controls were conducted using Pearson’s chi-squared test for the dichotomous variables and the t test for the continuous variables. Hardy–Weinberg equilibrium (HWE) was assessed using the Pearson’s chi-squared test. Unconditional logistic regression analyses were performed to test the association of each SNP with TB case/control status, adjusting for age and gender, under different genetic models (codominant, dominant, recessive and overdominant genetic models). All analyses were performed using the Statistical Package for the Social Sciences release 19.0 (SPSS Inc., Chicago, IL, USA). The association between haplotypes and TB susceptibility were also analyzed using unconditional logistic regression analyses, adjusting for age and gender, using SNPstats (http://bioinfo.iconcologia.net/snpstats/start.htm).

The meta-analyses were performed with STATA version 12.0 (StataCorp, College Station, Texas). Following the procedure reported by Thakkinstian *et al*.^26^, pairwise differences were used to determine the most appropriate genetic model for the meta-analysis. The procedure is as follows (assuming “A” as the risk allele compared with “a” allele; odds ratio (OR)_1_, OR_2_ and OR_3_ representing comparisons of AA vs. aa, Aa vs. aa and AA vs. Aa): (a) OR_1_ = OR_3_ ≠ 1 and OR_2_ = 1, a recessive model is suggested; (b) OR_1_ = OR_2_ ≠ 1 and OR_3_ = 1, a dominant model is suggested. (c) OR_2_ = 1/OR_3_ ≠ 1 and OR_1_ = 1, an overdominant model is suggested. (d) OR_1_ < OR_2_ < 1 and OR_1_ < OR_3_ < 1 (or OR_1_ > OR_2_ > 1 and OR_1_ > OR_3_ > 1), a codominant model is suggested.

*χ*^2^ based Q statistics and the *I*^2^ test were used to assess the between-study heterogeneity. These tests indicated a lack of heterogeneity between the discovery and replication sets, and therefore a fixed effects model (Mantel-Haenszel’s method) was used in the meta-analysis^27^.

Adjusted OR estimates and 95% CIs on a natural logarithmic scale were used to assess the strength of association between SNP and TB susceptibility. A *p* value < 0.05 was considered statistically significant in all the above statistical analyses except the between-study heterogeneity analysis.

## Results

### Discovery cohort

The discovery cohort was composed of 636 TB cases (324 males and 312 females, mean age = 36.77 ± 15.71) and 608 controls (302 males and 306 females, mean age = 37.14 ± 15.68) from the Chinese Han population. There was no significant difference in either age or gender between two groups, as the cases and controls were matched on these variables. No deviations from HWE were detected in the control subjects (*p* > 0.05). The genotype call rates were 99.4% for rs9932581, 98.2% for rs3794624 and 99.4% for rs4673.

The main results of the association between SNPs in *CYBA* and TB risk are presented in Table 1. After adjusting for age and gender, we found the A allele of the rs3794624 polymorphism to be a significant protective factor against TB in the Chinese Han population under two genetic models (GA vs. GG: OR 0.74, 95% CI 0.57–0.96, *p* = 0.03; GA vs. GG+AA: OR 0.73, 95% CI 0.56–0.95, *p* = 0.02). There was no significant association identified between rs9932581 T > C or rs4673 T > C polymorphisms and TB susceptibility under any genetic model.

Five *CYBA* haplotypes (GTG, GCG, ACG, ATG and GTA) were identified, with frequencies more than 0.03 (Table 2). We observed no significant differences of *CYBA* haplotype frequencies between TB and control groups, when adjusting for age and gender.

### Replication cohort

To validate the association of rs3794624 with TB risk we genotyped an independent cohort of Chinese Tibetans, including 613 TB cases (327 males and 286 females, mean age = 34.54 ± 13.87) and 603 controls (333 males and 270 females, mean age = 34.62 ± 13.84), with no difference in age and gender ratio between the groups. No deviation from HWE was detected in the control subjects (*p* > 0.05). The genotype call rate for rs3794624 was 99.9%.

The replication cohort showed strong supporting evidence for the association between rs3794624 polymorphism and TB risk. After adjusting for age and gender, we found the A allele of the rs3794624 polymorphism to be a significant protective factor against TB in the Chinese Tibetan population under three genetic models: overdominant (GA vs. GG+AA: OR 0.68, 95% CI 0.51–0.92, p = 0.01), heterozygous (GA vs. GG: OR 0.68, 95% CI 0.51–0.92, *p* = 0.01) and dominant (AA+GA vs. GG: OR 0.70, 95% CI 0.52–0.93, *p* = 0.02), as shown in Table 3.

### Meta analysis of the two included cohorts

**Figure 1:**
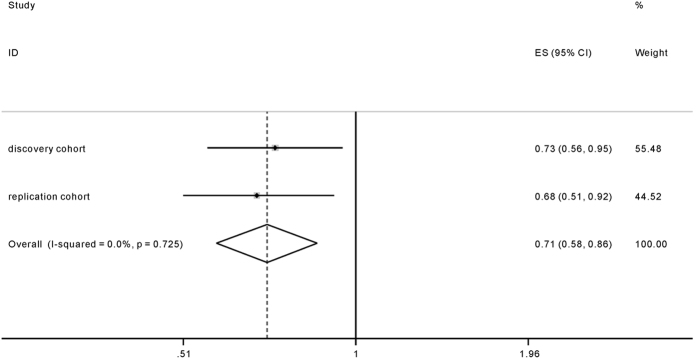
Forrest plot of the association between rs3794624 and TB risk under the overdominant model.

We included both discovery and replication cohorts in the following meta-analysis. The pooled OR_1_, OR_2_ and OR_3_ of rs3794624 genotypes with TB susceptibility are shown in Table 4. The Wald test showed that OR_2_ and OR_3_ were both significant (*p* = 0.001 and 0.04, respectively), while OR_1_ was not significant (*p* = 0.22). As a result, the overdominant model was then determined to be the most appropriate genetic model. The pooling analysis provided robust evidence for the association of rs3794624 GA heterozygote with decreased TB risk without any evidence of heterogeneity (GA vs. GG+AA: OR 0.71, 95% CI 0.58–0.86, *p* = 0.001), as shown in Fig. 1.

## Discussion

The NADPH oxidase complex is a key enzyme that catalyzes the production of ROS, which mediate oxygen-dependent killing of microorganisms, such as MTB, and also play a role in the injurious effects of oxidative stress on body tissues or organs^28^,^29^,^30^. Numerous reports demonstrating a high prevalence of BCG complications and TB risk in chronic granulomatous disease provide a strong rationale for the critical role of NADPH oxidase in controlling TB in humans^9^,^10^,^11^,^12^,^13^. The function of the NADPH oxidase complex is largely dependent upon its regulatory subunit p22phox (encoded by *CYBA*) which increase the stability the large subunit and undertakes the role as docking unit for the cytosolic factors^15^. Up until now, no other studies have address the role of polymorphisms in *CYBA* and TB susceptibility.

*CYBA* is located on the long arm of chromosome 16 (16q24) and spans approximately 8.5 kb, including six exons and five introns. Three SNPs (rs9932581 T > C, rs3794624 G > A and rs4673 T > C) were included in our initial association analysis. rs9932581 and rs4673 are both potential functional SNPs, based on *in silico* functional prediction and literature review^16^,^20^, but neither showed significant association with susceptibility to TB in our Chinese Han population. However, our study found an association between rs3794624 in *CYBA* with decreased TB susceptibility in two independent Chinese cohorts and in the meta-analysis.

**Figure 2:**
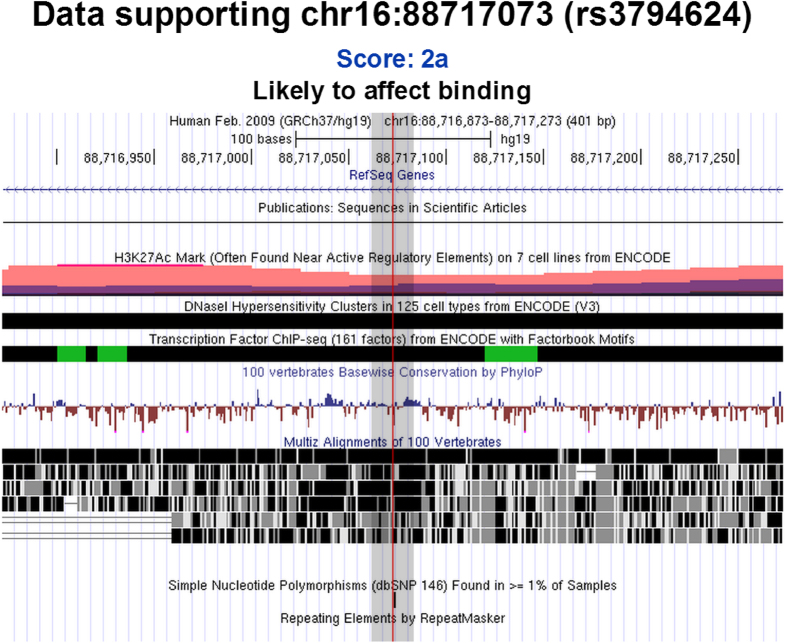
Functional prediction result of rs3794624 by Regulome DB.

rs3794624 has been associated with clinical outcomes such as peripheral arterial disease^19^ and breast cancer risk^22^ suggesting a possible functional role. In addition, both of the above-mentioned diseases are considered to be ROS-related. However, we did not find any study of this polymorphism that directly demonstrated a functional effect. rs3794624 is located in the first intron of *CYBA* and therefore may lead to the alternative splicing of the transcript or expression level change. The *in silico* functional prediction for rs3794624 suggests a potential functional significance of this polymorphism site. As shown in Fig. 2, rs3794624 G > A was predicted to be functional SNP in the Regulome DB (LSJU, Stanford, CA, USA) with a score of 2a, which is annotated as to be likely to affect binding with transcription factors and the supporting data of this locus were as follows: TF binding+matched TF motif+matched DNase Footprint+DNase peak. Thus, further investigation of the functional significance of rs3794624 is needed.

The association of rs3794624 with susceptibility to TB may also be due to LD with another polymorphism that has a functional effect. rs3794624 is in LD (r^2^ ≥ 0.7) with three other *CYBA* polymorphisms (rs33997949, rs13306296 and rs35601559) in the East Asian populations of the 1000 Genomes Project (http://www.1000genomes.org/). However, none of these three SNPs have any obvious functional significance.

As haplotypes are often more relevant than individual SNPs^31^, we further did haplotype analysis of *CYBA* SNPs in association with the risk of TB. However no statistically significant association was identified. This result may be ascribed to the fact that the protective effect of *CYBA* polymorphisms manifested as a heterozygote advantage over individuals carrying either homozygous genotype rather than the impact of a haplotype, which is the combination of alleles in different sites. We speculate that heterozygotes for rs3794624 are more fit in terms of natural selection because of their intermediate phenotype with respect to enzyme activity. As mentioned above, ROS released by NADPH oxidase complex is a double-edged sword in MTB immunity, as they can lead to the elimination of this pathogen but also result in injury due to oxidative stress. Therefore, the GA genotype of rs3794624 may lead to neither too much nor too little ROS production and provide the optimal protective effect in TB. In this regard, it is worth mentioning that Tarazona-Santos *et al*. conducted a resequencing analysis of *CYBA* in 102 individuals with different ethnicities, and reached similar conclusions to ours^32^. In their report, they found *CYBA* was characterized by high diversity and high frequency of common polymorphisms in Europeans, which was considered to be the result of balancing natural selection. Heterozygote advantage was speculated to be the biological mechanism underlying balancing natural selection. That is to say, heterozygous individuals producing an inter-mediate level of ROS, had a survival advantage during human evolution. Combining our research, we can infer that MTB-driven selective pressure may be one of the factors that promoted the balancing selection of *CYBA.*

Our research has several strengths. Firstly, to our knowledge, this is the first case-control study to investigate *CYBA* polymorphisms and TB susceptibility. Our study has shown rs3794624 as protective factor against TB in the Chinese population. Secondly, our study included two large cohorts (Chinese Han and Chinese Tibetan). The consistent results from two independent populations with different ethnic backgrounds considerably increase confidence that the association is not due to type I error.

Nevertheless, some limitations in this research should also be addressed. First, we did not perform functional validation of the associated SNP. As a result, the mechanism underlying the genetic association result is still unknown. Second, we did not correct our results for multiple testing, which may increase the chance of type I errors. However, the results from the replication cohort and the pooled analysis would survive such correction.

## Conclusions

In conclusion, we have demonstrated an association between the GA genotype of rs3794624 in *CYBA* with decreased TB susceptibility, which suggests new avenues for exploring the role of oxygen-dependent innate immunity against MTB in the development of TB. However, further analyses are needed to fully validate these findings in other ethnic populations and to reveal the potential function of this SNP.

## Additional Information

**How to cite this article**: Liu, Q. *et al*. Heterozygote Advantage of the rs3794624 Polymorphism in *CYBA* for Resistance to Tuberculosis in Two Chinese Populations. *Sci. Rep.* **6**, 38213; doi: 10.1038/srep38213 (2016).

**Publisher’s note:** Springer Nature remains neutral with regard to jurisdictional claims in published maps and institutional affiliations.

## Figures and Tables

**Table 1:** Association between *CYBA* SNPs and TB susceptibility in the discovery cohort.

SNP	Genetic model	Genotype	Controls, n (%)	TB patients, n (%)	OR (95% CI)^a^	*P* a value
rs9932581	Codominant	TT	209 (34.6%)	224 (35.4%)	1.00	—
		TC	289 (47.9%)	295 (46.7%)	1.00 (0.99–1.01)	0.37
		CC	106 (17.6%)	113 (17.9%)	1.17 (0.86–1.60)	0.31
	Dominant	TT	209 (34.6%)	224 (35.4%)	1.00	—
		CC+TC	395 (65.4%)	408 (64.6%)	0.97 (0.76–1.22)	0.77
	Recessive	TC+TT	498 (82.5%)	519 (82.1%)	1.00	—
		CC	106 (17.6%)	113 (17.9%)	1.02 (0.76–1.37)	0.88
	Overdominant	TT+CC	315 (52.1%)	337 (53.3%)	1.00	—
		TC	289 (47.9%)	295 (46.7%)	0.96 (0.76–1.20)	0.69
rs3794624	Codominant	GG	419 (70.5%)	471 (75.0%)	1.00	—
		GA	167 (28.1%)	140 (22.3%)	**0.74** (**0.57–0.96)**	**0.03**
		AA	8 (1.4%)	17 (2.7%)	1.38 (0.90–2.11)	0.14
	Dominant	GG	419 (70.5%)	471 (75.0%)	1.00	—
		AA+GA	175 (29.5%)	157 (25.0%)	0.80 (0.62–1.02)	0.08
	Recessive	GA+GG	586 (98.7%)	611 (97.3%)	1.00	—
		AA	8 (1.4%)	17 (2.7%)	2.05 (0.88–4.80)	0.09
	Overdominant	GG+AA	427 (71.9%)	488 (77.7%)	1.00	—
		GA	167 (28.1%)	140 (22.3%)	**0.73** (**0.56–0.95)**	**0.02**
rs4673	Codominant	GG	506 (83.6%)	549 (86.9%)	1.00	—
		GA	97 (16.0%)	82 (13.0%)	0.78 (0.57–1.07)	0.12
		AA	2 (0.3%)	1 (0.2%)	0.67 (0.20–2.23)	0.51
	Dominant	GG	506 (83.6%)	549 (86.9%)	1.00	—
		GA+AA	99 (16.4%)	83 (13.1%)	0.77 (0.56–1.06)	0.11
	Recessive	GG+GA	603 (99.7%)	631 (99.8%)	1.00	—
		AA	2 (0.3%)	1 (0.2%)	0.47 (0.04–5.20)	0.53
	Overdominant	GG+AA	508 (84.0%)	550 (87.0%)	1.00	—
		GA	97 (16.0%)	82 (13.0%)	0.78 (0.57–1.07)	0.13

Abbreviation: SNP, single nucleotide polymorphism; TB, tuberculosis; OR, odds ratio; CI, confidence interval.

^a^Adjusted for age and gender in a logistic regression model.

**Table 2:** Haplotype analysis of *CYBA* SNPs in association with the risk of TB.

rs3794624	rs9932581	rs4673	Frequency	OR (95% CI)^a^	*P* a value
G	T	G	0.479	1.00	*—*
G	C	G	0.303	0.95 (0.77–1.16)	0.59
A	C	G	0.081	1.02 (0.73–1.43)	0.90
A	T	G	0.062	0.71 (0.48–1.07)	0.10
G	T	A	0.045	0.81 (0.51–1.30)	0.39

Abbreviation: OR, odds ratio; CI, confidence interval.

^a^Adjusted for age and gender in a logistic regression model.

All haplotypes with frequencies <0.03 were ignored in the analysis.

**Table 3:** Association between *CYBA* rs3794624 and TB susceptibility in the replication cohort.

SNP	Genetic model	Genotype	Controls, n (%)	TB patients, n (%)	OR (95% CI)^a^	*P* a value
rs3794624	Codominant	GG	472 (78.4%)	514 (83.8%)	1.00	—
		GA	124 (20.6%)	92 (15.0%)	0.68 (0.51–0.92)	0.01
		AA	6 (1.0%)	7 (1.1%)	1.00 (0.99–1.01)	0.92
	Dominant	GG	472 (78.4%)	514 (83.8%)	1.00	—
		AA+GA	130 (21.6%)	99 (16.1%)	0.70 (0.52–0.93)	0.02
	Recessive	GA+GG	596 (99%)	606 (98.9%)	1.00	—
		AA	6 (1.0%)	7 (1.1%)	1.12 (0.37–3.37)	0.83
	Overdominant	GG+AA	478 (79.4%)	521 (85.0%)	1.00	—
		GA	124 (20.6%)	92 (15.0%)	0.68 (0.51–0.92)	0.01

Abbreviations: SNP, single nucleotide polymorphism; TB, tuberculosis; OR, odds ratio; CI, confidence interval.

^a^Adjusted for age and gender in a logistic regression model.

**Table 4:** Pairwise comparisons of rs3794624 genotypes and TB susceptibility before determining the best genetic model.

Study	OR1 (95% CI)^a^	OR2 (95% CI)^a^	OR3 (95% CI)^a^
Discovery cohort	1.38 (0.90–2.11)	0.74 (0.57–0.96)	2.55 (1.07–6.90)
Replication cohort	1.03 (0.59–1.78)	0.68 (0.51–0.92)	1.57 (0.50,4.88)
Overall	1.24 (0.88–1.73)	0.71 (0.59–0.87)	2.10 (1.02–4.32)

OR, odds ratio; CI, confidence interval.

^a^Adjusted for age and gender in a logistic regression model.
